# CMOS-Based Redox-Type Label-Free ATP Image Sensor for In Vitro Sensitive Imaging of Extracellular ATP

**DOI:** 10.3390/s22010075

**Published:** 2021-12-23

**Authors:** Hideo Doi, Tomoko Horio, Yong-Joon Choi, Kazuhiro Takahashi, Toshihiko Noda, Kazuaki Sawada

**Affiliations:** Department of Electrical and Electronic Information Engineering, Toyohashi University of Technology, Toyohashi 441-8580, Japan; horio-t@int.ee.tut.ac.jp (T.H.); choi@ee.tut.ac.jp (Y.-J.C.); takahashi@ee.tut.ac.jp (K.T.); noda-t@eiiris.tut.ac.jp (T.N.); sawada@ee.tut.ac.jp (K.S.)

**Keywords:** bio-imaging, label-free detection, adenosine triphosphate, redox-enzyme, potentiometric sensor array

## Abstract

Adenosine 5′-triphosphate (ATP) plays a crucial role as an extracellular signaling molecule in the central nervous system and is closely related to various nerve diseases. Therefore, label-free imaging of extracellular ATP dynamics and spatiotemporal analysis is crucial for understanding brain function. To decrease the limit of detection (LOD) of imaging extracellular ATP, we fabricated a redox-type label-free ATP image sensor by immobilizing glycerol-kinase (GK), L-α-glycerophosphate oxidase (LGOx), and horseradish peroxidase (HRP) enzymes in a polymer film on a gold electrode-modified potentiometric sensor array with a 37.3 µm-pitch. Hydrogen peroxide (H_2_O_2_) is generated through the enzymatic reactions from GK to LGOx in the presence of ATP and glycerol, and ATP can be detected as changes in its concentration using an electron mediator. Using this approach, the LOD for ATP was 2.8 µM with a sensitivity of 77 ± 3.8 mV/dec., under 10 mM working buffers at physiological pH, such as in in vitro experiments, and the LOD was great superior 100 times than that of the hydrogen ion detection-based image sensor. This redox-type ATP image sensor may be successfully applied for in vitro sensitive imaging of extracellular ATP dynamics in brain nerve tissue or cells.

## 1. Introduction

Adenosine 5′-triphosphate (ATP) is a well-known intracellular energy currency in all living cells and is necessary for many biological processes. ATP also plays a key role in the regulation of synaptic transmission as an extracellular signaling molecule from neurons and glial cells (non-neuronal types in the brain) in the central nervous system [1,2,3]. Moreover, it has been reported that extracellular ATP is closely related to neurodegenerative diseases and psychiatric disorders, including depression [4], but the detailed mechanism is not yet fully understood. Therefore, analysis of the spatiotemporal distribution of extracellular ATP is crucial for understanding brain function and various nerve diseases. 

ATP levels in living tissues, including the brain, have usually been measured by luminescence [5] or high-performance liquid chromatography (HPLC) [6] using microdialysis [7] samples. Although these analysis systems are very sensitive to ATP, they have a low temporal resolution, ranging from a few tens of seconds to several minutes, due to analytical samples being collected using the dialysis probe and no spatial resolution. As an alternative approach, various electrochemical ATP biosensors with enzymes immobilized on Pt disk [8], glassy carbon [9], Clark-type oxygen [10], and interdigitated gold [11] electrodes have been developed to measure ATP concentration. Further, amperometric microelectrode biosensors have been widely employed for extracellular signal-recording with direct real-time measurements in biological tissues [12,13,14,15] and cells [16,17]. These biosensors have a high time resolution and low detection limit but no spatial resolution because of their single-electrode local-point-sensing with a diameter of around 20–100 µm.

The spatiotemporal dynamics of extracellular ATP have been predominantly visualized using bioluminescence or fluorescence imaging. For ATP recognition, a luciferase probe [18], a fluorescent aptamer sensor [19], genetically encoded protein-based sensors [20,21], and ratiometric ATPOS sensors [22] have been developed. However, these methods require an any-labeling process for tissue or cells, and skillful techniques are also required. Additionally, chemical signaling in extracellular spaces may be inhibited by immobilizing ATP-recognition sensors on the cell membrane. Therefore, the development of a label-free method for visualizing extracellular ATP that does not require the labeling of tissue and cells is challenging. 

On the other hand, a potentiometric biosensor array based on the charge-transfer technique for imaging hydrogen ion (H^+^) movement in biological phenomena has been developed, which can be used to visualize the two-dimensional distribution of its dynamic behavior label-free and in real-time with high spatial resolution [23]. Using a developed sensor array for implantation in a living system, the visualization of proton dynamics in the brain has been demonstrated in vivo [24]. By immobilizing the target molecule-degrading enzyme on the ion-sensitive membrane of the sensor array, label-free real-time imaging of biomolecules, such as ATP [25] and acetylcholine (ACh) [26], is enabled. Recently, visualization of the spatiotemporal pattern of extracellular ATP has been demonstrated in biological tissues without any labeling [27]. However, the sensor output signal decreases due to the influence of pH-buffer components [28,29], which are generally used in in vitro environments, and the pH value in solution. The limit of detection (LOD) is also down to several hundreds of µM levels in principle, which limits the visualization of local ATP release and its spatiotemporal analysis.

In our group, a gold electrode-modified potentiometric redox sensor array for the detection of hydrogen peroxide (H_2_O_2_) has been developed [30,31,32]. The detection system mainly consists of horseradish peroxidase (HRP) and electron mediators. H_2_O_2_ can be detected as redox states of electron mediators on the surface of the gold electrode, and an LOD of 1 µM was achieved.

In this study, to overcome the limitation of the H^+^ detection-based ATP sensor, we fabricated an H_2_O_2_ detection technique-based redox-type label-free ATP image sensor with a 37 µm pitch and 128 × 128-pixels, in which three types of redox enzymes were immobilized using a polymer membrane, and the detection performance for ATP was investigated. Label-free real-time imaging of ATP distribution based on H_2_O_2_ detection techniques was demonstrated. We achieved a low detection limit with high sensitivity under the condition of 10 mM buffer actions at physiological pH. Redox ATP image sensors can be expected to be successfully applied for highly sensitive in vitro imaging of extracellular ATP from biological tissue or cells.

## 2. Materials and Methods

### 2.1. Materials and Chemicals

Glycerol kinase (GK) (Grade III, 20 U/mg-solid) and L-α-glycerophosphate oxidase (LGOx) (Grade III, 15 U/mg-solid) were purchased from Toyobo CO. Ltd. (Osaka, Japan). Horseradish peroxidase (HRP) (>250 units/mg-solid), ferrocenyl methanol (FcMeOH) (95%), poly-L-lysine (PLL) (Mw: 70,000~150,000), poly (sodium 4-styrene sulfonate) (PSS) (Mw: ~70,000), acetylcholine chloride (Ach) (~99%), gamma-aminobutyric acid (GABA) (>99%), and D-serine (>98%) were purchased from Sigma-Aldrich Inc. Sodium (St. Louis, MO, USA) 4-(2-hydroxyethyl) piperazine-1-ethanesulfonate (HEPES) (>99%) was purchased from Dojindo Laboratories. Adenosine-5′-triphosphate (98%), adenosine diphosphate (ADP) (97%), adenosine monophosphate (AMP) (99.3%), adenosine (Ado) (98%), L-glutamic acid sodium (99%), sodium chloride (99.5%), potassium chloride (99.5%), calcium chloride (95%), magnesium chloride, D-glucose (98%), potassium hexacyanoferrate (III) [K_4_Fe(CN)_6_] (99%), potassium hexacyanoferrate (II) [K_3_Fe(CN)_6_] (99.5%), glycerol (>99%), ethanol (95%), and sodium hydroxide solution were purchased from Wako Pure Chemical Industries, Ltd (Osaka, Japan). The FcMeOH solution was prepared using 95% ethanol, and all other reagents were prepared using ultrapure water (18.2 MΩ cm at 25 °C) produced with a Milli-Q water system (Tokyo, Japan).

### 2.2. The Measurement Principle of the Redox-Type Label-Free ATP Image Sensor

**Figure 1:**
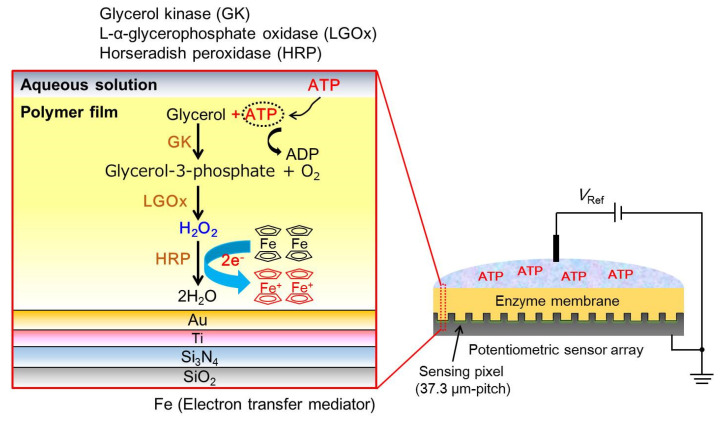
Schematic diagram showing the ATP sensing with redox reaction on a gold electrode. ATP image sensor is fabricated using enzymes GK, LGOx, and HRP entrapped in a polymer film.

To overcome the impact of the pH-buffering action on the sensor response, an H_2_O_2_ detection method based on the redox reaction was applied for the sensitive detection of ATP. A schematic diagram of the measurement principle for ATP sensing on a gold electrode-modified potentiometric sensor array is shown in Figure 1. The detection system for ATP consisted mainly of redox enzymes glycerol kinase (GK), L-α-glycerophosphate oxidase (LGOx), horseradish peroxidase (HRP), ferrocenyl methanol (FcMeOH) functioning as an electron-transfer-mediator, and the gold electrode modified to the sensor array which was fabricated by the standard CMOS process. In this study, the following enzyme reaction system for ATP was used.

(1) $$\mathrm{Glycerol}+\mathrm{ATP}\;\overset{\mathrm{GK}}{\to }\;\mathrm{Glycerol-}3\mathrm{-phosphate}+\mathrm{ADP},$$

(2) $$\mathrm{Glycerol-}3\mathrm{-phosphate}+O_{2}\;\overset{\mathrm{LGOx}}{\to }\;3\mathrm{-dihydroxyacetone}\;\mathrm{phosphate}+H_{2}O_{2},$$

(3) $$H_{2}O_{2}+2H^{+}+2\mathrm{FcMeOH}\;\overset{\mathrm{HRP}}{\to }\;2H_{2}O+2{\mathrm{FcMeOH}}^{+}$$

When the ATP is present as a target molecule in the solution, glycerol is catalyzed to glycerol-3-phosphate by GK. Then, it is further catalyzed to 3-dihydroxyacetone phosphate by LGOx, and H_2_O_2_ is generated during these enzymatic reactions. Additionally, generated H_2_O_2_ is catalyzed to H_2_O by HRP, where the FcMeOH as a redox mediator is oxidized by HRP and it changes to FcMeOH^+^ due to be removed electrons from FcMeOH. The surface potential *E* on the gold electrode is given as follows from the Nernst equation with a redox reaction.

(4) $$E=E^{o}+\frac{RT}{nF}ln\frac{\left[Fc^{+}\right]}{\left[Fc\right]}$$

where *E^o^* is the standard electrode potential, *R* is the universal gas constant, *T* is the absolute temperature, *n* is the number of electrons, and *F* is the Faraday constant. Therefore, the sensor output *V*_Out_ is shown in Equation (5), and it changes depending on the concentration of ATP in an aqueous solution.

(5) $$V_{\mathrm{Out}}=E$$

### 2.3. Sensor Fabrication

The redox-based label-free ATP image sensor was fabricated by immobilizing redox enzymes on a potentiometric sensor array with a spatial resolution of 37.3 µm. The device fabrication process was as follows. First, gold and titanium (Au/Ti: 20/5 nm) were deposited on a sensor array by an electron-beam physical vapor deposition process [30]. Subsequently, a 1 M NaOH solution was added dropwise onto the Au electrode surface and kept for 15 min. The surface of the sensor tip was rinsed with deionized water for 20 min and dried for 1 h at 25 °C. Subsequently, three types of redox-enzymes, GK, LGO, and HRP, were immobilized as an enzyme-functionalized membrane at the surface of the Au/Ti layered film by the mixed-layered technique with a poly-ion complex method [27]. A 10 µL-PSS solution (25 mM), 5 µL-GK solution (5 units: 1 unit/µL), 5 µL-LGO solution (5 units: 1 unit/µL), 5 µL-HRP solution (10.5 unit: 2.1 unit/µL), and 10 µL PLL solution (20 mM) was mixed in a microtube. Then, the mixed solution was added dropwise onto the sensing region and dried overnight at 4 °C. Ferrocene (FcMeOH) was used as an electron mediator. Additionally, an enzyme-free sensor was also prepared.

### 2.4. Measurement of Redox Response

**Figure 2:**
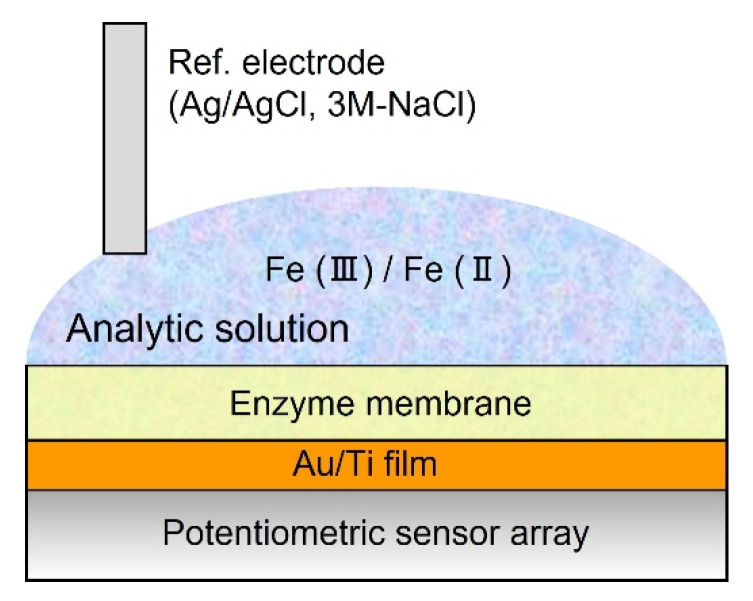
Schematic illustration of a cross−sectional view of measurement setup for redox response.

Prior to evaluation of the redox response, mixing solutions with oxidation-reduction species with concentration ratios of 99:1, 9:1, 1:1, 1:9, and 1:99 (potassium hexacyanoferrate(III) [K_4_Fe(CN)_6_]: potassium hexacyanoferrate(II) [K_3_Fe(CN)_6_]) were prepared. Subsequently, 90 µL of a mixed solution was placed on the sensor array and then replaced and the output voltage in response to the concentration dependence of the redox states was measured (see in Figure 2). A Ag/AgCl electrode filled with 3.3 M-NaCl in a plastic resin tube (LF-2, eDAQ Pty Co., Ltd., Nagoya, Japan) was used as a reference electrode. These aqueous solutions contained the following chemical components (in mM): NaCl 150, KCl 5, CaCl_2_ 2, MgCl_2_ 1, D-glucose 10, and HEPES 10 (pH 7.4).

### 2.5. ATP Measurement

**Figure 3:**
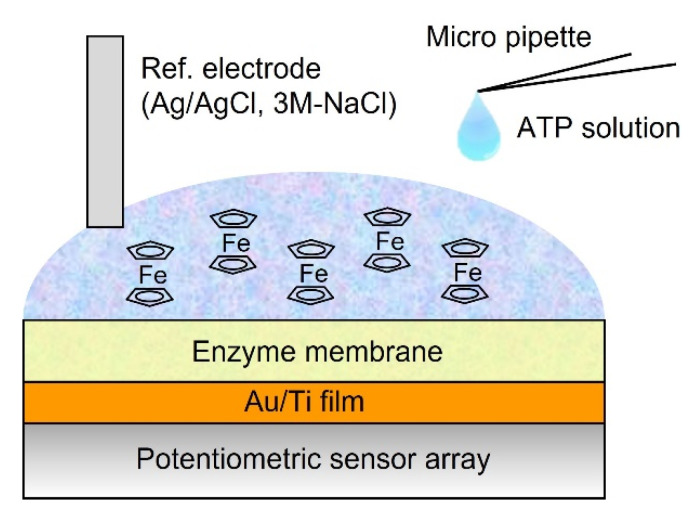
Schematic illustration of a cross−sectional view of experimental setup for ATP sensing.

Figure 3 shows a schematic of the ATP imaging system. A 90 µL buffer solution containing 500 µM FcMeOH and 2 mM glycerol was first set up on the sensor array. The reference electrode was fixed to the liquid surface of the buffer solution. Subsequently, a 10 µL ATP dissolved buffer solution was added to the buffer solution with a micropipette, and the final concentration was measured in the range of 10^−7^ to 10^−3^ M. HEPES was used as a pH buffering agent. The buffer and ATP-dissolved buffer solutions were composed of the following chemical components (in mM): NaCl 150, KCl 5, CaCl_2_ 2, MgCl_2_ 1, D-glucose 10, HEPES 10, glycerol 2 [13], and FcMeOH 0.5. These analytical solutions were controlled at pH 7.4 with NaOH. For the comparative study on the effect of pH buffering action on the sensor output, ATP measurements at concentrations of both 1 mM and 100 mM HEPES were conducted, and the LOD in each buffer condition was compared. Furthermore, the LOD for the redox-based ATP image sensor was also compared to that of the existing sensor device based on the H^+^ detection technique using the ATP-degrading enzyme apyrase.

## 3. Results and Discussion

### 3.1. Fabricated Sensor

**Figure 4:**
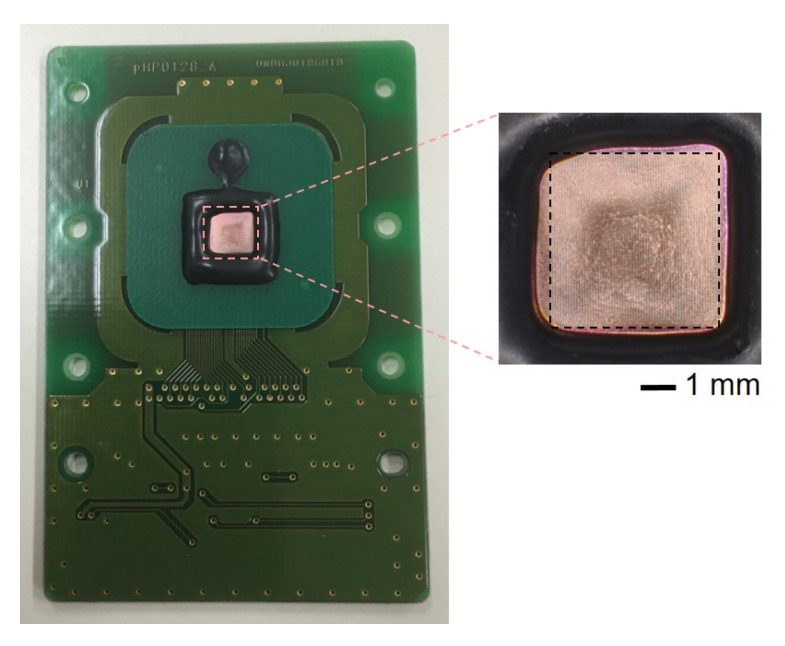
Optical microscopic image of a redox-type label−free ATP image sensor. The sensor area for ATP detection is indicated with a black dash line.

Figure 4 shows the optical microscopic images of a redox-type label-free ATP image sensor and enzyme membrane formed on the sensor array. As can be seen in Figure 4, it was confirmed that the enzyme membrane was formed on the sensing region. The membrane thickness was approximately 1 µm.

### 3.2. Redox Response

**Figure 5:**
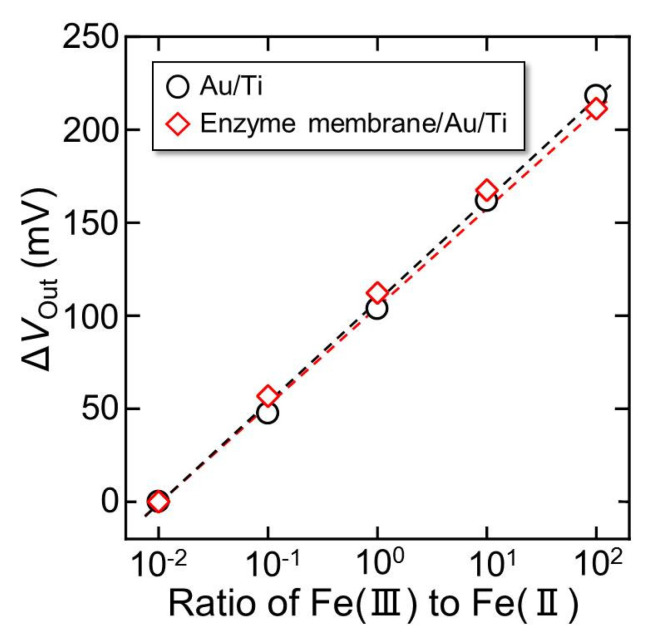
Δ*V*_Out_ of the sensor plotted against the concentration ratio of Fe (III) to Fe (II).

To confirm that the enzyme-immobilized sensor functions as a redox sensor, the potential change in the redox response was measured and compared to that of the sensor without an enzyme membrane. Figure 5 shows the relationship between the output voltage change (Δ*V*_Out_) of the sensor and the concentration ratio of Fe (III) to Fe (II). The Δ*V*_Out_ for the enzyme-immobilized sensor changed linearly with increasing the Fe (III) concentration to Fe (II) and showed an almost equivalent response to that of the bare electrode. The sensitivity of the enzyme-immobilized sensor was 53.4 mV/decade, whereas the sensitivity of the bare electrode was 55.1 mV/decade, and the difference among samples was 1.7 mV/decade. Because the variation in the sensitivity between these samples occurred at approximately 3 mV/decade, the decreasing sensitivity of the enzyme-immobilized membrane may be negligible. Therefore, it was confirmed that the fabricated sensor functions as a redox sensor with a suitable sensitivity to detect the redox response even when the membrane is formed. 

### 3.3. Output Characteristics of the Redox-Type ATP Image Sensor

**Figure 6:**
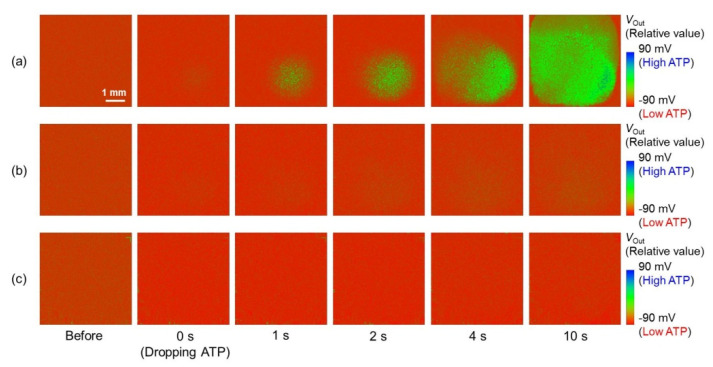
Temporal change in the output images after the addition of ATP solution. ATP concentration is 1 mM. (**a**) The output image to ATP measured by the enzyme−immobilized sensor, (**b**) the output image of the enzyme−immobilized sensor measured in no glycerol, and (**c**) the output image of an enzyme−free sensor.

Figure 6 shows the output image change when the ATP solution was added to the buffer solution filled on the sensor. The output voltage (*V*_Out_) of the sensor array is expressed as relative values over a range of 90 mV, as shown by the color scale. As shown in Figure 6a, the output image of the enzyme-immobilized sensor clearly changed after the addition of ATP, and the *V*_Out_ in the sensor array increased gradually (color change from red to green). On the other hand, the output image of the enzyme-free sensor hardly changed over the entire sensing region. Additionally, the output image change for the enzyme-immobilized sensor was not observed in the no-glycerol condition; it was observed only in the presence of enzymes and glycerol. Next, the temporal change in *V*_Out_ change (Δ*V*_Out_) in the sensor array of each output image shown in Figure 4 was analyzed.

**Figure 7:**
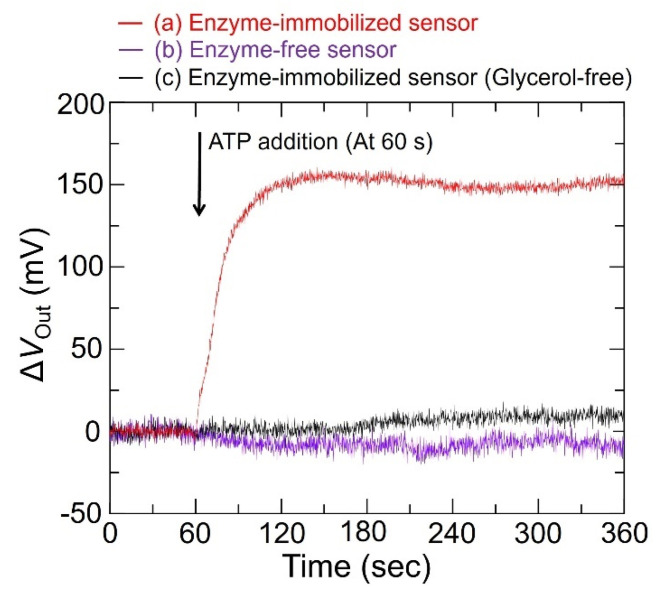
Characteristics investigation of output response after 1 mM–ATP was added to the sensor. (a) Typical Δ*V*_Out_–time curve to ATP measured by the enzyme–immobilized sensor, (b) time response of an enzyme−free sensor, and (c) time response of enzyme−immobilized sensor measured in no glycerol. Working buffer is 10 mM−HEPES at pH 7.4.

Figure 7 shows the time course of a single pixel in the sensor response to ATP. After the dropwise addition of ATP at 60 s, the Δ*V*_Out_ for the enzyme-immobilized sensor increased to approximately 150 mV and reached saturation. Conversely, Δ*V*_Out_ for the enzyme-free sensor and the enzyme-immobilized sensor in the case of no-glycerol conditions hardly changed after the addition of ATP, and significant differences between each sample were observed. These results show that the change in Δ*V*_Out_ for the enzyme-immobilized sensor originated from an increase in the concentration of H_2_O_2_ generated by the corresponding enzyme reactions. Therefore, real-time imaging of ATP diffusion based on the H_2_O_2_ detection technique with a redox reaction was demonstrated. 

**Figure 8:**
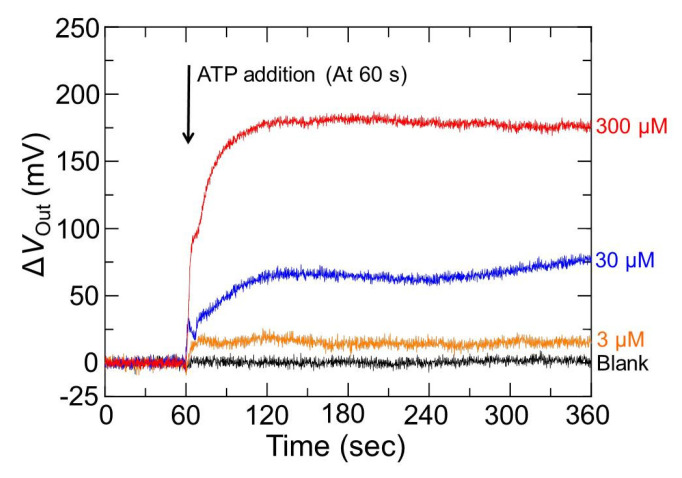
Temporal changes of the sensor response to the different ATP concentrations and blank concentration. All experiments were performed in 10 mM−HEPES at pH 7.4.

**Figure 9:**
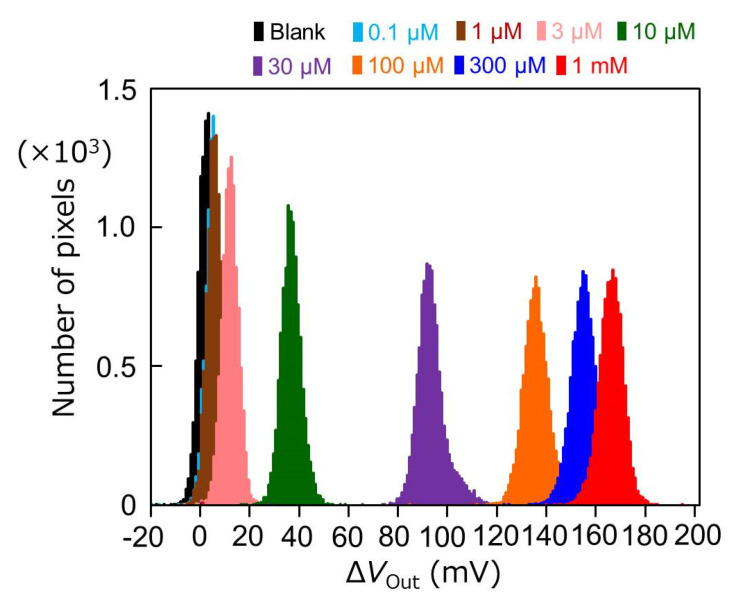
Output distribution of the potential difference between before and after the addition of ATP.

Figure 8 shows the time dependence of Δ*V*_Out_ for different ATP concentrations measured by the enzyme-immobilized sensor. The sensor response for the enzyme-immobilized membrane changed depending on the concentration of ATP, where no signal response was observed in the blank concentration as ATP-free. These results are attributed to the increase in the amount of H_2_O_2_ per unit time generated in the membrane due to an increase in the enzymatic reaction rate, which increases with increasing ATP concentration. On the other hand, the time response of the sensor decreased with an increase in the ATP concentration of the analytical solution. This probably reflects ATP diffusion into the enzyme membrane and the reaction time of the corresponding enzymes until the glycerol is degraded to H_2_O. Next, the output distribution in the sensor array for different ATP concentrations was investigated using the following formula, which is defined as

$$\Delta V_{\mathrm{Out}}=V_{\mathrm{Out},\mathrm{at}\;360\;s}-V_{\mathrm{Out},\mathrm{initial}\;(\mathrm{at}\;50\;s)}$$

where *V*_Out, at 360 s_ is the output response at 360 s measured by each pixel of the sensor array after the addition of ATP, and *V*_Out__, initial (at 50 s)_ is the initial value in each pixel before ATP addition. As shown in Figure 9, the output distribution of the enzyme-immobilized sensor with various concentrations of ATP showed a Gaussian distribution, and its peak shift was confirmed.

**Figure 10:**
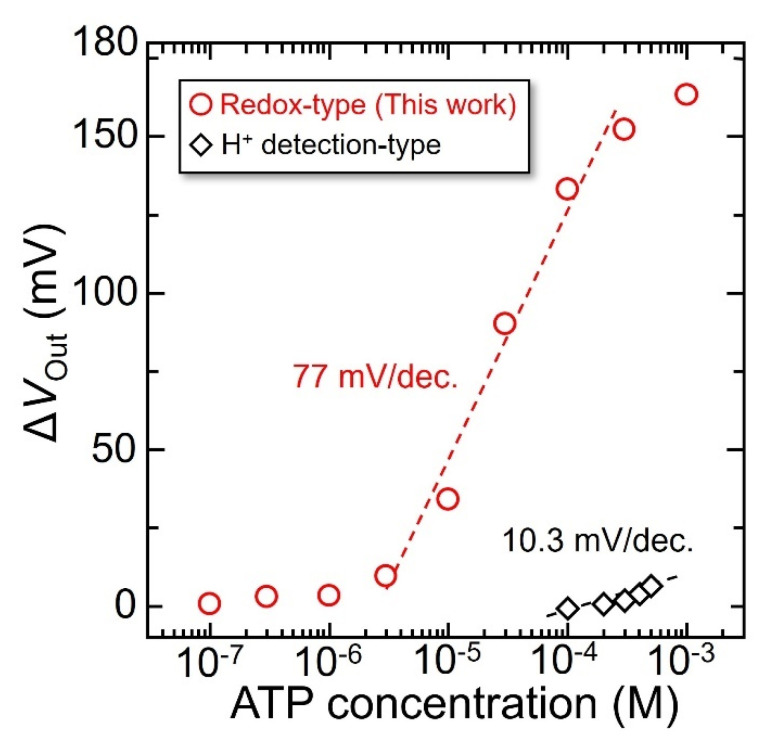
Concentration dependence of enzyme-immobilized sensor on ATP concentration. Working buffer is 10 mM−HEPES, at pH 7.4.

Figure 10 shows the Δ*V*_Out_ plotted as an average value of the histogram shown in Figure 7 for the ATP concentration. The Δ*V*_Out_ for the redox-type ATP image sensor with the enzyme membrane increased linearly in the range of 3–300 µM, and the sensitivity was 77 ± 3.8 mV/decade (*R*^2^ = 0.974). The lowest detectable ATP concentration was 2.8 µM (*S*/*N* = 3) under the measurement condition of a 10 mM working buffer at 7.4 pH (LOD, defined as the concentration equivalent of three times the standard deviation of the sensor output signal before ATP addition). This exhibited a lower LOD than that of the already existing imaging device, in which ATP was detected based on the changes in the concentration of H^+^ generated by the enzymatic reaction of ATP-degrading enzyme apyrase (see in Figure 8). The difference in the LOD between these methods is discussed in the next section. In addition, the enzyme-immobilized sensor showed high repeatability (R.S.D. < 5%, *N* = 4), and it was found exhibited that the redox-type image sensor could detect ATP with relatively high repeatability and could be used for repeated measurements several times. However, the effect of the number of immobilized enzymes and membrane thickness on the sensor response is still unclear; optimizing the enzyme-entrapped membrane is necessary for high-speed detection of ATP.

### 3.4. The Influence of pH Buffering Action on the Limit of Detection

In order to evaluate the influence of pH buffering action such as in the biological experiment, ATP measurement was conducted by controlling the concentration of HEPES with pH buffering action, and the LOD for the redox-type ATP image sensor was compared to that of the imaging device with the conventional H^+^ detection method, as described in Section 3.2. 

**Figure 11:**
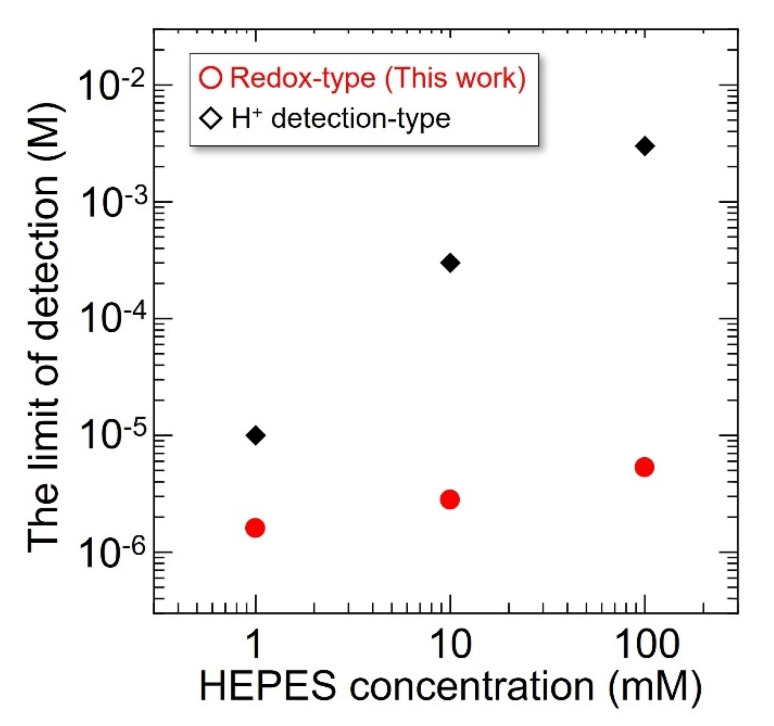
The detection limit of the ATP image sensor plotted against the various concentrations of HEPES at pH 7.4.

Figure 11 shows the relationship between the LOD of the sensor and the HEPES concentration. The LODs were calculated from each standard curve for ATP determination. As shown in Figure 10, the change in the LOD for the redox-type ATP image sensor with the H_2_O_2_ detection technique with respect to HEPES concentration was not significant. In contrast, in the case of the sensor with the H^+^ detection technique, the LOD significantly decreased with increasing concentration of HEPES. This mainly originates from the amount of H^+^ generated by the enzymatic reaction of ATP with apyrase that is strongly reduced by the pH buffering action corresponding to HEPES concentration, as described previously [33], indicating that the pH buffering action in an aqueous solution considerably affects the chemical reaction system for ATP detection. Therefore, it was confirmed that the redox-type ATP image sensor with the H_2_O_2_ detection technique exhibited a lower LOD than that of the sensor with the H^+^ detection technique under the condition of a 10 mM HEPES buffer, such as in the biological experiment. 

Table 1 shows a comparison of the performance characteristics of the ATP image sensor. Regarding ATP imaging, a 37.3 µm-pitch and 16,384 pixels of the potentiometric sensor array on which ATP was measured by converting to H^+^ using ATP-degrading enzyme apyrase have been reported so far. Notably, in Ref [34], although the LOD was achieved on the order of 1 µM, the buffer concentration and pH in aqueous solution were 1 mM and 8.0, respectively. Thus, in the case of such measurement conditions, in principle, it is expected that the actual LOD and sensitivity in in vitro experiments are significantly decreased in terms of the concentration of the buffer component and pH value in the aqueous solution. This is because the biological experiment is conducted under the condition of a buffer solution near several tens of mM and pH 7.4. In contrast, the sensitivity and LOD for the redox-type ATP image sensor using redox enzymes GK, LGOx, and HRP showed high performance with high sensitivity and low LOD compared to existing methods under the condition of 10 mM HEPES at physiological pH of 7.4. 

Table 2 shows the comparison of analytical characteristics of the electrochemical biosensors for ATP determination. LOD and the linear working range of the proposed sensor are comparable with other biosensors. Biosensor based on the GOD/HEX has a short linear range but an excellent LOD. Additionally, enzyme-based amperometric microelectrode biosensors are used in some biological experiments due to their low cost, simplicity, and ease of use, and real-time monitoring of extracellular ATP has been demonstrated in biological tissue [12,13,14,15]. However, these biosensors have only a single-point electrode for ATP measurement, that is, it is difficult to reduce the size to several μm and increase the density because of the amperometric detection method. Importantly, amperometric sensor devices cannot be adapted for the spatiotemporal analysis of extracellular ATP distribution. In contrast to probe electrode-based reports, the fabricated redox-type ATP biosensor array has a reasonable spatial resolution of 37.3 µm and low LOD with high sensitivity, which has excellent potential for highly sensitive in vitro imaging of extracellular ATP and its spatiotemporal analysis. In a recent study, high-density ion-image sensor with a pitch resolution of several micrometers, which was fabricated by CMOS process technology, has been realized [35,36]. Applying our developed ATP detection technique to a CMOS sensor array with high spatial resolution and high-speed operation, it also has the potential for high spatiotemporal imaging of extracellular ATP. Therefore, our novel ATP image sensor, which combines the redox-based H_2_O_2_ detection technique with a CMOS image sensor technology, is expected to be applied for in vitro imaging as an effective method for bioimaging of extracellular ATP from nerve tissue or cells in biological experiments.

### 3.5. Selectivity for ATP

**Figure 12:**
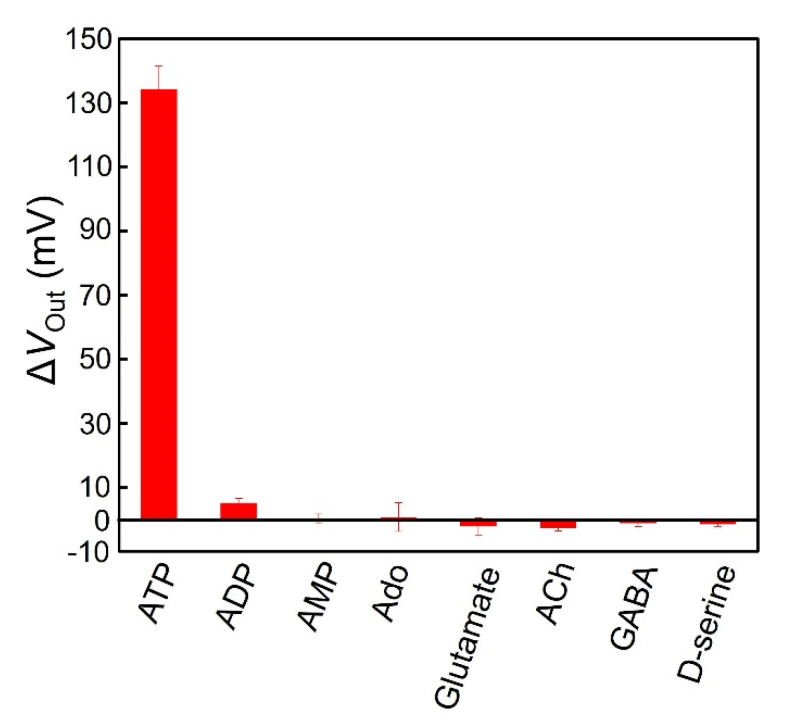
The selectivity of ATP detection. The concentration of ATP and other chemical substances is 100 µM. All experiments were performed in 10 mM−HEPES at pH 7.4. An error bar shows the standard deviation (*n* = 3).

To examine the selectivity of the redox-type ATP image sensor with the enzyme-immobilized membrane for ATP, some analogous molecules such as ADP, AMP, and adenosine (Ado), and representative chemical molecules in the brain, such as glutamate, acetylcholine (ACh), γ-aminobutyric acid (GABA), and D-serine, were investigated. As shown in Figure 12, when ATP, ADP, AMP, Ado, glutamate, ACh, GABA, and D-serine were added to the sensor array, only the addition of ATP produced an obvious sensor response, suggesting high selectivity for ATP. Because of the high specificity of GK for glycerol and because its enzyme reaction is promoted in the presence of ATP, H_2_O_2_ cannot be produced in the case of other chemical molecules, indicating the high selectivity of our novel ATP imaging device based on the H_2_O_2_ detection technique for ATP. Additionally, because the actual extracellular concentration levels in the brain are expected to be several tens of µM or less, the proposed redox-type ATP image sensor allows for the accurate determination of ATP.

## 4. Conclusions

For sensitive imaging of extracellular ATP in the brain, a CMOS-based, redox-type, label-free ATP image sensor was fabricated by the immobilization of enzymes GK, LGO, and HRP at the surface of the gold electrode deposited on the potentiometric sensor arrays having a spatial resolution of 37.3 µm, and the detection performance for ATP sensing was investigated. Based on H_2_O_2_ detection with corresponding redox reactions, label-free sensitive imaging of ATP diffusion was demonstrated for the first time and exhibited a low detection limit and high sensitivity under the pH buffer level used in a biological experiment. The proposed redox ATP image sensor based on H_2_O_2_ detection techniques was successfully applied for sensitive in vitro imaging and spatiotemporal analysis of extracellular ATP from brain nerve tissue or cells. It can also be used for other biological samples. Due to the semiconductor chip becoming very cheap due to mass-production, it is expected that the bioimage sensor chip will be able to be obtained for under 10 dollars in future years.

## Figures and Tables

**Table 1:** Comparison of sensing performance of the image sensor for ATP detection and its experiment condition.

Bio-Recognition	Pixel-Pitch	Pixel Number	Sensitivity	LOD	HEPES	pH	Reference
H^+^-Apyrase	37.3 µm	16,384	No data	100 µM	1 mM	8.0	[25]
H^+^-Apyrase	37.3 µm	16,384	15.5 mV/dec	10 µM	1 mM	7.4	[27]
H^+^-Apyrase	37.3 µm	16,384	37.8 mV/mM	1.3 µM	1 mM	8.0	[34]
GK-LGOx-HRP	37.3 µm	16,384	77 ± 3.8 mV/dec	2.8 µM	10 mM	7.4	This work

GK: glycerol kinase, LGOx: L-α-glycerophosphate oxidase, and HRP: horseradish peroxidase.

**Table 2:** Comparison of analytical characteristics of the proposed ATP image sensor with other electrochemical biosensors for ATP measurement.

Approach	Bio-Recognition	LOD	Linear Range	Spatiotemporal Analysis	Reference
Amperometric	GOD-HEX	9.9 ± 3.2 nM	0.25–4 µM	×	[12]
Amperometric	GK-G3PO	200 nM	200 nM–50 µM	×	[13]
Amperometric	HEX	15 µM	15–300 µM	×	[11]
Amperometric	SHL-G6PDH-HEX	3 µM	5 µM–4 mM	×	[10]
Potentiometric	GK-LGOx-HRP	2.8 µM	3–300 µM	◯	This work

GOD: glucose oxidase, HEX: hexokinase, GK: glycerol kinase, G3PO: glycerol-3-phosphate oxidase, SHL: salieylate hydroxylase, G6PDH: glucose-6-phosphate dehydrogenase, LGOx: L-α-glycerophosphate oxidase, and HRP: horseradish peroxidase.
